# Thirty‐day recurrence after ischemic stroke or TIA

**DOI:** 10.1002/brb3.1108

**Published:** 2018-09-17

**Authors:** Andrej Netland Khanevski, Anna Therese Bjerkreim, Vojtech Novotny, Halvor Næss, Lars Thomassen, Nicola Logallo, Christopher E. Kvistad

**Affiliations:** ^1^ Department of Clinical Medicine University of Bergen Bergen Norway; ^2^ Department of Neurology Haukeland University Hospital Bergen Norway; ^3^ Norwegian Health Association Oslo Norway; ^4^ Centre for Age‐related Medicine Stavanger University Hospital Stavanger Norway; ^5^ Department of Neurosurgery Haukeland University Hospital Bergen Norway

**Keywords:** cerebrovascular disease, ischemic stroke, readmission, stroke etiology, stroke recurrence

## Abstract

**Background:**

Incidence of recurrent stroke is highest within 30 days after the initial ischemic stroke (IS) or TIA, but knowledge about early recurrence is lacking. We aimed to identify etiological groups with highest risk of early recurrence and assess how the TOAST classification identified index stroke etiology.

**Methods:**

Medical records of 1874 IS and TIA patients in the Bergen NORSTROKE registry were retrospectively reviewed for identification of recurrent IS or TIA within 30 days after index IS or TIA. Stroke etiology was determined by review of electronical medical journals. Logistic regression was used to calculate odds ratios (OR) for 30‐day recurrence.

**Results:**

Thirty‐three patients (1.8%) were readmitted with recurrent IS or TIA within 30 days after index stroke. By using TOAST, 12 patients were initially classified with stroke of unknown etiology (SUE). Etiologies behind recurrent IS or TIA were after the recurrent episode identified as extracranial large artery atherosclerosis (LAA) in 14 patients (42.4%), intracranial arterial pathology in seven patients (21.2%), active malignancy in six patients (18.2%), and cardio embolism in four patients (12.1%). Small vessel occlusion and SUE were the causes in one patient each. Logistic regression showed that patients with stroke of other determined etiology (SOE) and LAA had increased risk of 30‐day recurrence (OR = 9.72, 95% CI 1.84–51.3, *p* < 0.01 and OR = 4.36, 95% CI 2.01–9.47, *p* < 0.01, respectively).

**Conclusion:**

Patients with LAA and SOE had increased risk of recurrent IS or TIA within 30 days. TOAST was inadequate at identifying exact etiologies behind recurrent stroke at index event.

## 1 INTRODUCTION

The purpose of diagnostic evaluation of acute stroke is identification of the causal etiology behind it, in order to reduce the risk of further strokes with appropriate secondary prevention. Recurrent stroke is associated with increased disability and mortality compared to the index stroke (Petty et al., 1998; Sacco, Wolf, Kannel, & McNamara, 1982). And even with appropriate prevention, the risk of recurrence after ischemic stroke (IS) and transient ischemic attack (TIA) is high, especially in the early phase after stroke (Lovett, Coull, & Rothwell, 2004; Sacco, Shi, Zamanillo, & Kargman, 1994). Paradoxically, many large studies on recurrent stroke incidence have excluded events occurring within the first month leaving a lack of data regarding early recurrence (Coull & Rothwell, 2004). Studies on 30‐day recurrence are scarce and use varying definitions of recurring events. A meta‐analysis found a pooled cumulative risk of 3.1% for 30‐day recurrence, but risk estimates of 30‐day recurrence vary greatly (Mohan et al., 2011). Moreover, when classifying etiology by TOAST, around 40% of patients will end up with an undetermined or cryptogenic etiology when an apparent cause is lacking or identification of multiple competing etiologies (Hart et al., 2014). Most recurrent IS is of the same type as the index episode, except for lacunar IS, which seems especially true for early recurrence (Jones, Sen, Lakshminarayan, & Rosamond, 2013; Yamamoto & Bogousslavsky, 1998). This implicates that patients with the highest risk of early recurrence may not obtain a specific etiology and thus not receive the most effective secondary prevention. A better understanding of causes behind early recurrence is therefore of utmost importance, as it may optimize prevention and thereby reduce morbidity and mortality.

We aimed to determine the etiology in patients rehospitalized with recurrent IS or TIA within 30 days after index stroke onset. We also aimed to identify etiological groups with the highest risk of early recurrence and see how TOAST identified the etiologies at index.

## 2 METHODS

All patients older than 18 years of age admitted with IS or TIA to the stroke unit at the Department of Neurology, Haukeland University Hospital, between July 1, 2007, and December 31, 2013, were registered in the Bergen NORSTROKE registry. IS was defined as an episode of neurologic deficit lasting >24 hr where magnetic resonance imaging (MRI) or computed tomography (CT) showed infarctions related to the clinical findings (Johnson et al., 1995). TIA was defined as a clinical diagnosis of transient focal cerebral dysfunction lasting shorter than 24 hr with no objective evidence of brain infarction on imaging (Johnson et al., 1995). All patients were investigated with brain imaging (CT and/or MRI), carotid ultrasound, and 24‐hr electrocardiographic monitoring. Trial of Org 10172 in Acute Stroke Treatment (TOAST) criteria was used for etiology classification. TOAST classifies stroke as caused by large‐artery atherosclerosis (LAA), cardio embolism (CE), small vessel occlusion (SVO), other determined cause (SOE), or stroke of undetermined cause (SUE; Adams et al., 1993).

Recurrent IS or TIA was identified by review of electronic medical records from Haukeland University Hospital unit and the other eight hospitals under the Western Norway Regional Health Authority, up to 30 days after completing evaluation and being discharged alive following the index IS or TIA from our stroke unit. New focal episodes from the same arterial territory during the initial hospitalization were considered as progression of acute stroke and not recurrence. We excluded patients who died before discharge and patients discharged to palliative care. We did also not include intracerebral hemorrhage or subarachnoid hemorrhage as recurrence. TOAST etiology for the index IS or TIA was obtained from the Bergen NORSTROKE registry. Intracranial lesions were studied separately because it is challenging to discriminate between embolic and atherosclerotic intracranial arterial pathology, especially in the early phase after the initial stroke (Lee, Oh, Bang, Joo, & Huh, 2004).

After the recurrent IS or TIA, a new etiological evaluation by review of supplemental clinical and imaging data was performed by two doctors with experience in stroke medicine (ANK and HN). A possible common cause for both episodes was determined.

The study was approved by the Western Regional Ethics Committee with written informed consent obtained from all patients or their legally authorized representatives.

### 2.1 Statistics

Chi‐squared test was used to assess baseline characteristics for categorical variables. Students’ *t* test or Mann–Whitney's *U* test was used to investigate continuous variables and expressed as mean with standard deviation (*SD*) or as median with interquartile range (IQR) if skewed. Backward stepwise regression was used to investigate the significant factors associated with 30‐day recurrence. Stata/SE 15.1 (Stata Corp LLC) were used for the analyses.

## 3 RESULTS

We identified 1874 patients who were discharged alive from our stroke unit after their index IS or TIA during the inclusion period, of whom 1668 with IS and 206 with TIA. CE was the most frequently determined cause of IS or TIA (*n* = 604, 32.3%), followed by LAA (*N* = 245, 13.0%), SVO (*N* = 205, 10.9%) and SOE (*N* = 32, 1.7%) during index admission. An etiology could not be determined in 42.1% of the patients.

A total of 33 patients, 28 with IS and five with TIA, (1.8%) were readmitted within 30 days after index stroke onset. Baseline characteristics are shown in Table 1. Patients with recurrence more frequently had a history of peripheral artery disease, carotid endarterectomy, a shorter length of stay (LOS) and were more often discharged home without assistance after the index event. There were no differences in age, gender, mRS at discharge, NIHSS score on admission, or treatment with IV thrombolysis between patients with 30‐day recurrent IS or TIA and patients with no recurrent stroke. Five patients that experienced new focal episodes from the same vascular territory during index admission, along with two patients that suffered early recurrence with hemorrhagic stroke, were excluded from the recurrence group.

**Table 1:** Baseline characteristics of population

Characteristics	30‐day recurrence *N* = 33	No 30‐day recurrence *N* = 1841	*p*
Age (years) mean ± *SD*	74.4 ± 11.5	73.2 ± 13.6	0.63
Sex (male)	17 (51.5)	1,019 (55.4)	0.66
mRS score, median (IQR)	1 (0, 2)	2 (0, 3)	0.06
NIHSS score, median (IQR)	1 (0, 4.5)	2 (1, 5)	0.09
BI score, median (IQR)	100 (100, 100)	100 (70, 100)	0.02
Index etiology (TOAST)			<0.01
Atherosclerosis	13 (39.4)	232 (12.6)	<0.01
Cardioembolism	5 (15.5)	599 (32.5)	0.03
Small vessel disease	1 (3.0)	204 (11.1)	0.14
Other determined	2 (6.1)	30 (1.6)	0.05
Undetermined	12 (36.6)	776 (42.2)	0.52
Comorbidities			
Peripheral artery disease	7 (21.2)	129 (7.0)	<0.01
Diabetes	6 (18.2)	275 (14.9)	0.61
Angina pectoris	3 (9.1.4)	256 (13.9)	0.43
Myocardial infarction	4 (12.1)	265 (14.4)	0.71
Hypertension	23 (69.7)	1,031 (56.0)	0.12
Atrial fibrillation	8 (18.2)	318 (18.4)	0.89
Prior/current smoking	21 (63.6)	1,066 (57.9)	0.39
Treatment			
IV thrombolysis	7 (21.2)	299 (16.2)	0.44
Carotid endarterectomy	5 (15.2)	64 (3.5)	<0.01
Length of index admission, median (IQR)	5 (2, 7)	6 (3, 10)	0.01
Discharged to			0.01
Home	22 (66.7)	985 (53.7)	0.13
Home nursing	2 (6.1)	193 (10.5)	0.41
Rehabilitation	0	152 (8.3)	0.09
Nursing home	5 (15.2)	444 (24.2)	0.23
Other department	4 (12.1)	59 (3.2)	<0.01

Note

BI, Barthel Index; IQR, interquartile range; mRS, modified Rankin Scale; NIHSS, National Institutes of Health Stroke Scale; *SD*, standard deviation.

Data are expressed as *n* (%) unless specified.

Etiology as classified by the TOAST classification after the index event is shown in Table 1. LAA was the most frequent index etiology in patients with 30‐day recurrence (*n* = 14), followed by intracranial vascular lesions (*n* = 8), CE (*n* = 5), SOE (*n* = 2), SVO (*n* = 1), and SUE (*n* = 12). Median time from index stroke to recurrence was 14 days (IQR 9, 20). No patient had TIA at both events. Ten patients self‐reported previous TIA symptoms. An MRI was performed in 29 of 33 patients at both index and recurrent event. Two patients had no MRI performed at any event.

After evaluating new clinical, radiological, and other investigative data after the recurrent event, specific etiology causing both index and recurrent IS or TIA could be identified in all but one patient (Table 2). Recurring stroke made a more specific classification in all except one patient with SUE from the index event possible. LAA of extracranial arteries was considered cause of both index and recurrent stroke in 14 patients (42.4%) instead of 13 at index. Six patients had experienced TIA symptoms previous to index admission. Eleven of 14 patients had a carotid stenosis >50% ipsilateral to the affected hemisphere. Five of 11 patients underwent carotid endarterectomy (CEA), four of them after the second stroke, and one after the first, with a median time from index stroke to surgery of 12 days (IQR 8, 12). In one patient, an intima flap following CEA occluded the carotid artery causing a new IS within 30 days after index.

**Table 2:** Specific etiologies for patients after recurrence

Total (*n* = 33)	0–30 days	%
Readmitted patients, total	33	
Large‐artery atherosclerosis (LAA)	14	42.4
Intracranial pathology	7	21.2
Cardioembolism (CE)	4	12.1
Small vessel occlusion (SVO )	1	3
Other demonstrated cause (SOE)	6	18.2
Stroke of unknown etiology (SUE)	1	3

Seven patients (21.2%) had intracranial vascular lesions responsible for recurrent stroke in the same vascular territory: three with stenosis of the middle cerebral artery (MCA), two with intracranial carotid stenosis, one advanced intracranial atherosclerosis, and one with an MCA aneurysm.

Six patients (18.2%) had recurrent IS or TIA because of hypercoagulability caused by underlying active cancer. Only one patient was recognized as having cancer‐related stroke and classified as SOE at index event. Three were classified as CE and two as SUE.

Atrial fibrillation was the final cause of stroke in four patients (12.1%) instead of five classified at index. Two patients classified as CE at index were diagnosed with cancer‐associated stroke at recurrence. Of three CE patients, two patients were prescribed anticoagulants after the index event and one after the second event.

Small vessel occlusion was the cause of recurrent stroke in one patient. Table 3 shows results from the logistic regression analyses where 30‐day recurrence vs. no 30‐day recurrence is the dependent variable. IS or TIA due to SOE and LAA had significantly increased risk of 30‐day recurrence compared to other etiologies, (HR = 9.72, 95% CI 1.84–51.3, *p* < 0.01 and HR = 4.36, 95% CI 2.01–9.47, *p* < 0.01, respectively). Patients with a longer length of stay ≥6 days or peripheral artery disease also had significantly higher risk of 30‐day recurrence.

**Table 3:** Logistic regression analysis with 30‐day recurrence as dependent variable

	Odds Ratio	95% CI	*p*
Age, year	1.01	0.98–1.04	0.53
Female	1.39	0.66–2.95	0.38
NIHSS at admittance	0.99	0.91–1.08	0.87
Length of stay >6 days	0.90	0.82–0.99	0.03
LAA	4.36	2.01–9.47	<0.01
SOC	9.72	1.84–51.30	<0.01
Peripheral artery disease	2.61	1.03–6.60	0.04

Note

LAA, Large artery atherosclerosis; mRS, modified Rankin Scale; SOC, Stroke of other demonstrated cause; tPA, tissue plasminogen activator (intravenous thrombolysis).

## 4 DISCUSSION

We found that 1.8% of our patients was readmitted with a recurrent IS or TIA within 30 days. There is scant information on stroke recurrence in Norway, but our rate is low compared to many large international incidence studies (Bravata, Ho, Meehan, Brass, & Concato, 2007; Lovett et al., 2004; Sacco et al., 1994; Smith, Frytak, Liou, & Finch, 2005). Most of these studies included patients dating several decades back. Since then, improved secondary prevention may have reduced incidence of recurrent stroke and other vascular events (Hong, Yegiaian, Lee, Lee, & Saver, 2011). Our low 30‐day recurrence rate may reflect this progress in stroke medicine. Another explanation might be different definitions of recurrence.

Large artery atherosclerosis had the highest risk of 30‐day recurrence in our study, as previously reported by other studies (Lovett et al., 2004; Petty et al., 2000; Sacco, 1997). Considering that CEA is highly effective for reducing recurrent stroke in selected patients with stenosis of the internal carotid artery (Rothwell et al., 2003), knowing the risk of early recurrence is important for the stroke clinician in order to select and time patients for surgery. This is well illustrated in our study, as four of five patients in our study found eligible for CEA, suffered a recurrent stroke before surgery. On the other hand, peri‐ and postoperative complications after CEA may happen, as one patient in our study suffered a recurrent stroke due to a postoperative intimal flap. Patients with atherosclerotic lesions affecting extracranial arteries supplying the brain other than the internal carotid are also susceptible to atherosclerotic and occlusive disease causing recurrent stroke, but these patients have no proven benefit of surgical intervention. TOAST criteria require an intra‐ or extracranial stenosis of >50% to classify a stroke as LAA, a criterion originating from the NASCET study in which carotid stenoses were graded from angiograms (North American Symptomatic Carotid Endarterectomy Trial Collaborators et al., 1991). Since then, growing evidence shows that even in nonstenotic intra‐ or extracranial arteries, high‐risk atherosclerotic plaques may be the culprit of IS or TIA due to artery‐to‐artery embolization (Brinjikji et al., 2016; Kim & Kim, 2014). These cases nevertheless often end up being classified as cryptogenic, which may explain why patients with LAA in our study were initially classified as SUE, as more modern imaging techniques like emboli detection monitoring and contrast‐enhanced ultrasound were not routine practice in our stroke unit in the study period.

In our study, a significant part of patients with recurrence had intracranial vascular lesions causing recurrent IS or TIA. Intracranial stenosis is less common in a more Caucasian population as ours, but still has a high risk of causing recurrent stroke (Holmstedt, Turan, & Chimowitz, 2013). As intracranial vessels may become stenotic or occluded due to both atherosclerotic and embolic disease, we did not etiologically group them as LAA, but as a separate group. Spontaneous recanalization of emboli in the intracranial arteries will often occur, but may happen both in the acute phase and days and weeks after the initial stroke (Rha & Saver, 2007). As a result of this, it may be difficult to discriminate between LAA and CE as cause of an intracranial lesion in the first weeks and months after stroke. All patients with intracranial etiology in our study were investigated for both extracranial atherosclerotic and cardiac pathology, and received medical therapy and risk factor management without any mechanical intervention. Patients with severe intracranial vascular pathology in our population also seem prone to early recurrence.

Active cancer was the suspected cause of both index and recurrent stroke or TIA in six (18.2%) patients. Cancer cells secrete procoagulant factors that may induce a hypercoagulable state causing ischemic stroke and other thromboembolic complications (Bick, 2003). In clinical practice, cancer‐related stroke is considered rare and difficult to diagnose (Grisold, Oberndorfer, & Struhal, 2009). In our study, several of these patients were initially classified as CE because of infarcts in multiple vascular territories. Patients with cancer‐related coagulopathy may be prone to early recurrence and our findings suggest that in patients with recurrent IS or TIA within 30 days, it may be warranted to screen for a malignancy, especially in patients with embolic infarctions. Our numbers are very small though and need to be confirmed in larger studies.

Patients with CE and SVO had the lowest rate of 30‐day recurrence with around 0.5% each. In other studies, early CE stroke recurrence varies between 1% and 10% (Arboix & Alio, 2010). Many of these studies were however performed before anticoagulation was implemented as standard practice. Studies investigating the effect of aspirin vs. low molecular‐weight heparin in the early phase have generally shown no benefit of anticoagulation, suggesting that early re‐embolization is rare (Paciaroni, Agnelli, Micheli, & Caso, 2007). Recently published data from our stroke registry show no correlation between time to MRI and frequency of multiple acute cerebral infarcts in cardio embolic IS, suggesting that cardiogenic emboli seem to happen as a shower and not successively (Novotny, Khanevski, Thomassen, Waje‐Andreassen, & Naess, 2017).

SVO also is reported to have low recurrence rates (Jones et al., 2013; Lovett et al., 2004; Petty et al., 2000; Sacco, 1997). Although the exact pathology of stroke due to SVO is still under debate, the process leading to stroke is thought to be intrinsic to the small vessels (Wardlaw, 2005). This would mean that there are fewer sources of thrombus or emboli compared with non‐SVO stroke. A small meta‐analysis found a lower early risk of recurrence in patients with SVO compared to non‐SVO stroke (Jackson et al., 2009). In our data, only one patient suffered recurrent stroke due to SVO within 30 days.

This study has some limitations. This study is not prospective and the estimation of the recurrence rate is based on patients who from our stroke unit who sought medical care and were readmitted to our stroke unit or other hospital under the Western Norway Regional Health Authority. Furthermore, the single‐hospital design may cause sampling bias and reducing the number of patients with recurrent stroke, thus limiting the generalizability of our findings. However, we do not have data if patients were hospitalized in other regions of Norway or abroad. We cannot exclude that some patients may have suffered recurrent IS or TIA without a readmission to the hospital, even if we have a very low admission threshold in suspected acute stroke. On the other hand, it provides us with precise clinical data as compared to studies using administrative databases for identification of both recurrent stroke and their causes. In addition, we deviated from the TOAST classification in assessment of intracranial vascular lesions.

A strength of this study is that it investigates a general stroke population by review of medical records which gives a real‐life perspective on early recurrent stroke. Our single‐hospital design gives a high possibility of identifying most recurrent stroke cases that were hospitalized in our hospital or neighboring hospitals in Western Norway. Furthermore, it adds insight into patients groups not previously well studied for 30‐day recurrence, such as patients with intracranial vascular lesions and cancer.

In conclusion, most recurrent early IS and TIA cases are caused by LAA of extracranial arteries and intracranial atherosclerotic or embolic disease. TOAST identified only a proportion of patients accurately at the index event. Patients with recurrent stroke that lack conventional risk factors may be considered for cancer screening.

## CONFLICT OF INTEREST

None declared.
